# Common statistical errors in systematic reviews: A tutorial

**DOI:** 10.1002/cesm.70013

**Published:** 2025-01-29

**Authors:** Afroditi Kanellopoulou, Kerry Dwan, Rachel Richardson

**Affiliations:** ^1^ Methods Support Unit, Evidence Production and Methods Directorate Cochrane London UK; ^2^ Department of Hygiene and Epidemiology Faculty of Medicine University of Ioannina Ioannina Greece; ^3^ Department of Clinical Sciences Liverpool School of Tropical Medicine Liverpool UK

## Abstract

The aim of this article is to present the most common statistical errors in meta‐analyses included in systematic reviews; these are confusing standard deviation and standard error, using heterogeneity estimators for choosing between a common‐effect and random‐effects model, improper handling of multiarm trials, and unnecessary and misinterpreted subgroup analyses. We introduce some useful terminology and explain what authors can do to avoid these errors and how peer reviewers can spot them. We have also developed a micro‐learning module to provide practical hands‐on tutorial.

## Graphical abstract

The aim of this article is to present the most common statistical errors in meta‐analyses included in systematic reviews; these are confusing standard deviation and standard error, using heterogeneity estimators for choosing between a common‐effect and random‐effects model, improper handling of multiarm trials, and unnecessary and misinterpreted subgroup analyses. We introduce some useful terminology and explain what authors can do to avoid these errors and how peer reviewers can spot them. We have also developed a micro‐learning module to provide practical hands‐on tutorial. Common Statistical Errors micro learning module

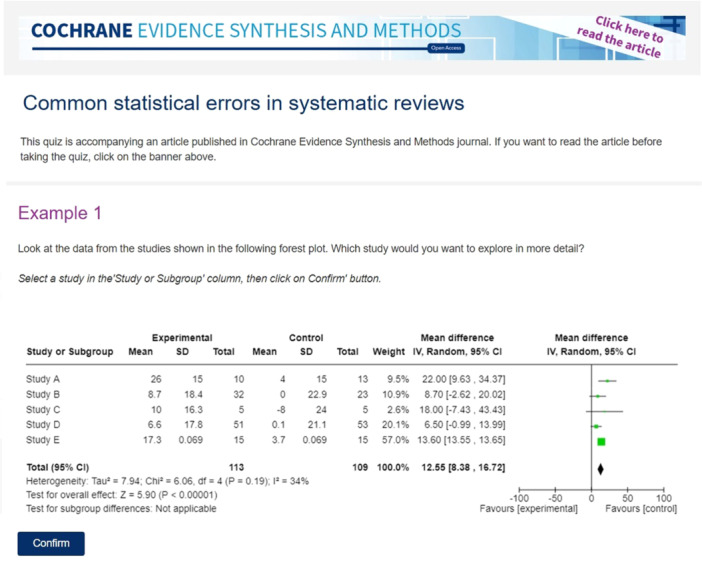

## 1 DATA ENTRY ERRORS: CONFUSING STANDARD DEVIATION (SD) AND STANDARD ERROR (SE)

### 1.1 Problem

When a meta‐analysis is conducted using continuous outcome data, authors may need to extract summary outcome measurements per study arm at one or several time points. These measurements are usually expressed as sample means (μ) and SDs. We call the measure a sample mean as it is an estimate taken from a sample of the whole population. The SD is a measure of how much the individual data points vary from the sample mean, that is, how widely the data are dispersed. For example, if the outcome variable Y has six values (2, 2, 3, 4, 4, 5), its mean and SD are calculated as follows:

$$\mu =\frac{2+2+3+4+4+5}{6}=\frac{20}{6}=3.3,\;SD=\sqrt{\frac{{\left(2-3.3\right)}^{2}+{\left(2-3.3\right)}^{2}+{\left(3-3.3\right)}^{2}+{\left(4-3.3\right)}^{2}+{\left(4-3.3\right)}^{2}+{\left(5-3.3\right)}^{2}}{6}}=1.2.$$

The arm‐specific means and SDs are used to calculate an overall mean difference (or standardized mean difference) which is used in the meta‐analysis and expresses the difference in outcome means between arms.

Confusing SD with another metric, that is SE, is one of the most common data entry errors in systematic reviews. While the SD describes the actual variance in the data, the SE is an estimate of what the variance might be if we took repeated samples from the whole population. The SE is a measure of how much the mean of the repeated samples would vary from the true mean of the population. The more samples that we take, the closer our estimate of the mean will be to the true mean and the lower the SE will be, as we are estimating the population mean more precisely. Furthermore, the SE will always be smaller than the SD; in the previous example, SE is equal to $\frac{1.2}{\sqrt{6}}=0.49$, a smaller value than 1.2.

### 1.2 TAKE‐HOME MESSAGE FOR AUTHORS AND PEER REVIEWERS

Review authors should have a basic knowledge of the subject being studied and the methodology followed in the individual reports, especially study design and statistical analysis. They should perform careful data extraction following predefined instructions, for example, reading carefully the sections describing the descriptive measures used and paying attention to the labels/footnotes of tables and figures. Data should also be extracted more than once from every report by independent data extractors to control for potential data entry errors [1].

On the other hand, peer reviewers should be able to spot metrics that stand out, for instance, extremely large or small SDs in relation to the other studies included in the meta‐analysis. Furthermore, it is strongly recommended to double‐check the analysis results against the report for the study assigned the biggest weight in a meta‐analysis, or even completely at random.

## 2 ERRORS IN THE INTERPRETATION OF HETEROGENEITY: USING HETEROGENEITY ESTIMATORS TO CHOOSE BETWEEN COMMON‐EFFECT AND RANDOM‐EFFECTS MODEL

### 2.1 Problem

The two commonly used meta‐analytical models are the common‐effect and random‐effects model. In brief, a common‐effect model assumes that all individual effect estimates are estimating the same underlying true effect, known as the “common‐effect” assumption. The random‐effects model is based on the assumption that the individual studies are estimating different, although related, effects. When there is evident heterogeneity among the studies, these two approaches provide different results.

Heterogeneity is a vague term which describes any kind of variability among studies included in a systematic review, namely clinical, methodological and statistical between‐study heterogeneity [2]. Regarding the latter, numerous methods have been proposed in the scientific literature according to which, review authors can identify and quantify the magnitude of statistical heterogeneity in a meta‐analysis. One of the most popular is the Cochran's Q statistic, according to which, Q values higher than the critical point for a given significance level (usually, *α* = 0.10) enable us to conclude that there is statistically significant between‐study heterogeneity. A second popular option is the *I* ^2^ index developed by Higgins and Thompson [3] which quantifies the proportion of total variability due to true between‐study heterogeneity (rather than chance). Thresholds for the interpretation of the *I* ^2^ index in a meta‐analysis have also been proposed [4], according to which, if *I* ^2^ index ranges between:

- 0% and 40%, then heterogeneity might not be important.
- 30% and 60%, then heterogeneity is moderate.
- 50% and 90%, then heterogeneity is substantial.
- 75% and 100%: then heterogeneity is considerable.

### 2.2 TAKE‐HOME MESSAGE FOR AUTHORS AND PEER REVIEWERS

Despite their widespread use and straightforward interpretation, statistical tests for heterogeneity should never guide the choice between a common‐effect and a random‐effects meta‐analysis. Q statistic and *I* ^2^ suffer from low power when the studies included in the meta‐analysis are few in number or have small sample sizes. This means that while a statistically significant result provides evidence of the presence of heterogeneity, a nonsignificant result must not be considered as evidence of no heterogeneity. In fact, choosing between a common‐effect and random‐effects model should mainly rely upon authors' consideration of whether studies are similar enough on all aspects that could importantly modify the magnitude of intervention effect, such as populations (e.g., participant baseline characteristics), interventions (e.g., dose and frequency of administration), outcomes (e.g., measurement scale, follow‐up time), and study designs, among others.

## 3 UNIT‐OF‐ANALYSIS ERRORS: STUDIES CONTRIBUTING TO A META‐ANALYSIS MORE THAN ONCE

### 3.1 Problem

**Figure 1:**
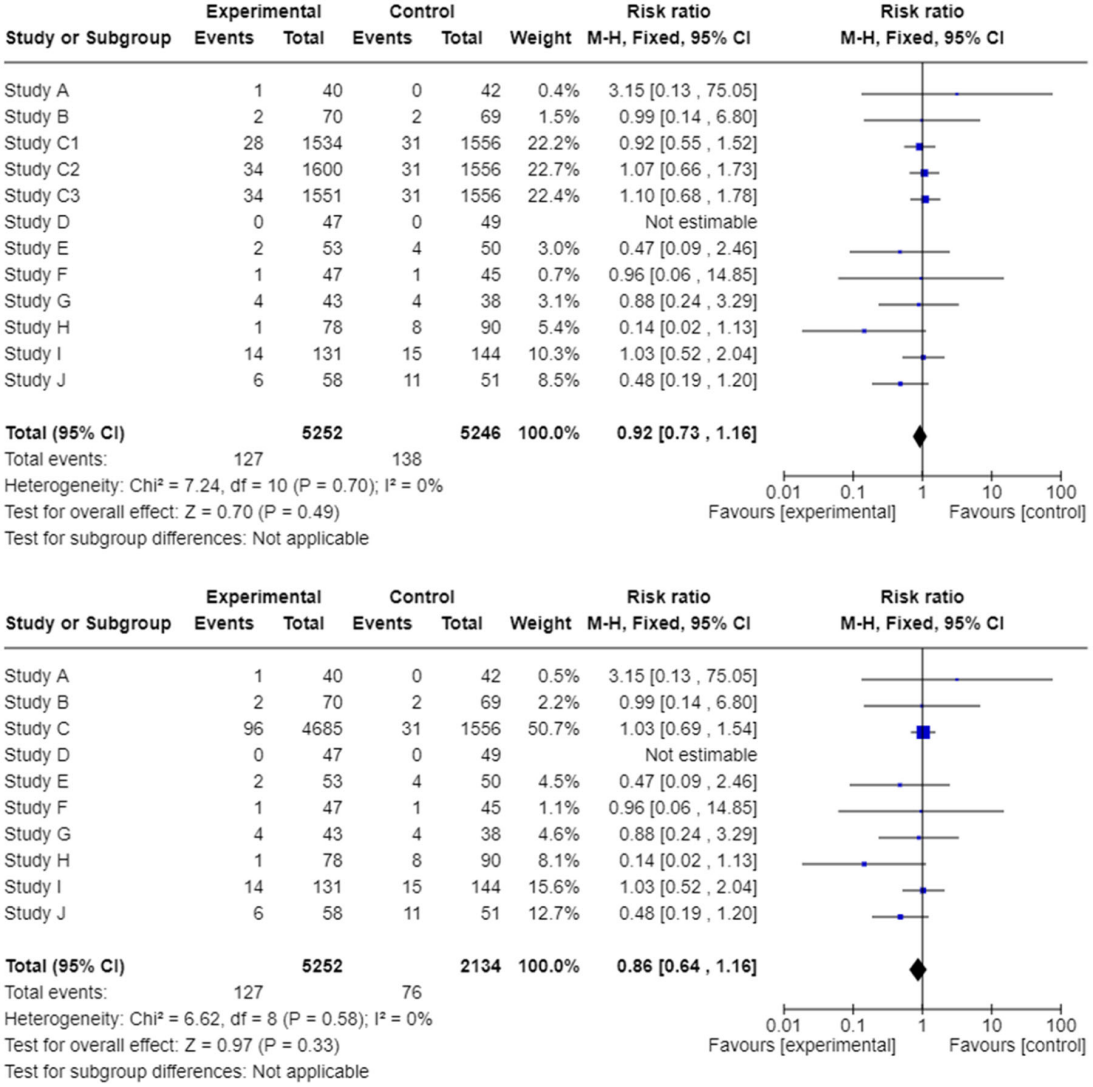
Forest plots of a hypothetical meta‐analysis comparing experimental and control arm. In the upper graph, a four‐arm randomized clinical trial (Study C) is included thrice causing a unit‐of analysis error due to triple counting of the participants in the control arm. In the lower graph, the issue is resolved by summing up the number of events and participants in all three experimental arms.

Studies with more than one intervention or comparator arm, known as multiarm studies, are quite common in biomedical research. Depending on the nature of the research question, not all comparisons of a multiarm study might be of interest for a systematic review. For instance, review authors might be keen to investigate dietary interventions which aim to reduce adverse events in cancer survivors (dichotomous outcome). If they identify a trial with three arms, say dietary counseling plus standard care, exercise plus standard care, and standard care alone, then the exercise arm is not relevant to the review so the review authors can ignore this arm and treat the study as a standard two‐arm trial. On the other hand, if review authors wish to investigate lifestyle interventions in general, then both intervention arms are relevant to their research question and may be included in a single meta‐analysis. In such a scenario, the comparator arm (standard care alone) will be included twice in the meta‐analysis which is usually referred to as “double counting” of participants and known as “unit‐of‐analysis” error. As a consequence, the corresponding meta‐analytic weights might be inflated, possibly causing significant errors in the estimation of the effect of the intervention (Figure 1).

### 3.2 TAKE‐HOME MESSAGE FOR AUTHORS AND PEER REVIEWERS

If review authors wish to include studies with multiple, correlated, comparisons, approaches to mitigate a unit‐of‐analysis error are available for both dichotomous and continuous outcomes [5, 6]. Review authors can combine data to create a single pair‐wise comparison; in the previous example, review authors can combine data from dietary counseling and exercise arms and then compare them together with standard care alone. Quite often, review authors choose to split the “shared” arm (in our case, the comparator arm) into two or more arms with smaller sample sizes and include two or more independent comparisons in the meta‐analysis. However, this approach violates the randomization of participants in the comparator arm. Correlated effect estimates can be included in separate meta‐analyses or in the same meta‐analysis if review authors make proper adjustments for their correlation, for instance, by calculating a weighted average along with a variance (weight) for the study [5]. Finally, review authors might consider performing a network meta‐analysis [7].

## 4 ERRORS IN SUBGROUP ANALYSES: UNNECESSARY SUBGROUPING AND INCOMPLETE INTERPRETATION

### 4.1 Problem

In a subgroup analysis, all data included in the meta‐analysis are split into subgroups, based on patient (such as sex and age category) or trial characteristics (such as geographical location), and a meta‐analysis is then performed on each subgroup. Usually, subgroup analysis is used to explore possible sources of between‐study heterogeneity or to answer research questions with respect to specific participants, types of interventions or types of study [2]. However, in a recent review including 52 Cochrane reviews, authors highlighted that only a small percentage of subgroup analyses results were sufficiently interpreted, meaning that most review authors usually face significant challenges when designing and conducting subgroup analyses [8].

### 4.2 TAKE‐HOME MESSAGE FOR AUTHORS AND PEER REVIEWERS

At first, review authors need to carefully consider whether subgroup analyses (defined a priori in their review protocol) have a clinical meaning and are feasible. Although it is difficult in practice, it is essential to limit the number of covariates proposed and include those that are clinically relevant, to protect against false positive conclusions (increased Type I error) [2]. With regard to the interpretation of subgroup analysis results, review authors need to take into account a number of criteria [9]. In particular, they should consider if the corresponding covariate statistically significantly modifies treatment effect. Usually, when the *p* value of the subgroup effect is <0.10, then this is indicative of a statistically significant subgroup effect. Additionally, review authors should discuss the direction and magnitude of the subgroup effect estimates as well as the extent of the residual heterogeneity within each subgroup. An important aspect is having similar distributions on the number of studies and participants between subgroups. The Cochrane Handbook recommends at least 10 studies to be included in the meta‐analysis so that any investigation of heterogeneity through subgroup analysis will produce meaningful findings [2]. The biological plausibility of the interaction should be commented too [9], meaning that existing research, such as animal studies or studies investigating similar interventions, should provide evidence of a potential subgroup effect. Finally, the importance of the interaction (or lack of interaction) as well as the possibility of confounding should be taken into consideration. In other words, review authors should wonder whether any differences between subgroup effect estimates can actually impact clinical decision‐making or may be due to confounding rather than effect modification.

## 5 FURTHER READING AND ONLINE CONTENT

We have developed a micro‐learning module, including a short quiz with feedback, to illustrate the common errors covered in this tutorial [10]. Additional material is also provided in the learning module “Common errors: Interpretation of statistical results” developed by Cochrane Training. This can be found here.

## AUTHOR CONTRIBUTIONS

**Afroditi Kanellopoulou**: Conceptualization; methodology; project administration; writing—original draft; writing—review and editing. **Kerry Dwan**: Conceptualization; supervision; writing—review and editing. **Rachel Richardson**: Conceptualization; supervision; writing—review and editing.

## CONFLICT OF INTEREST STATEMENT

Afroditi Kanellopoulou and Rachel Richardson are employed by Cochrane. Kerry Dwan is a former employee of Cochrane.

## 1 PEER REVIEW

The peer review history for this article is available at https://www.webofscience.com/api/gateway/wos/peer-review/10.1002/cesm.70013.

## Data Availability

Data sharing is not applicable to this article as no data sets were generated or analyzed during the current study. The data used in this article is “hypothetical.”
